# A case of emergency reconstructive surgery following facial destructive gunshot wounds: clinical and medico-legal assessments

**DOI:** 10.1186/s12245-023-00572-3

**Published:** 2023-12-20

**Authors:** Luca Tomassini, Giuliano Ascani, Paolo Mancini, Claudio Cacaci, Roberto Scendoni

**Affiliations:** 1 International School of Advanced Studies, University of Camerino, Camerino, Italy; 2 Department of Maxillofacial Surgery, Spirito Santo Hospital, Pescara, Italy; 3 Department of Law, Institute of Legal Medicine, University of Macerata, Macerata, Italy

**Keywords:** Facial gunshot wounds, Reconstructive surgery, Medico-legal assessment, Permanent impairments, Cost of firearm injuries

## Abstract

**Background:**

Facial gunshot wounds present a complex challenge to both medical professionals and victims with significant physical, psychological, and economic implications for those who suffer these types of injuries. Reconstructive surgery offers satisfactory aesthetic and functional outcomes, improving a patient’s quality of life. In these cases, the surgical procedure may encompass additional phases beyond those initially identified based on the type of wound and the extent of tissue destruction. As a result, each case necessitates thorough evaluation to determine an appropriate strategy. Nonetheless, it is worth noting that the outcomes achieved in terms of both aesthetics and functionality in this domain have the potential to be excellent.

**Case presentation:**

A 66-year-old man attempted suicide with a shotgun, causing severe facial injuries and fractures. He had a history of depression and was taken to the emergency department promptly. CT scans revealed brain and facial bone injuries, and he underwent surgery to control bleeding and tracheostomy. Postoperative recovery was successful. The patient’s condition stabilized, and he was discharged after 10 days. Follow-up visits showed gradual healing. Despite an offer for further facial reconstruction, he declined, satisfied with the achieved results.

**Conclusions:**

The present case report is intended to support the argument that effective facial reconstruction should be considered in the medico-legal assessment. It could be beneficial to introduce a new classification system and personalized evaluation methods with careful consideration given to treatment costs (which can be very high) and expected results. Since reconstructive surgery modifies damage and impacts the long-term costs of permanent impairments, its inclusion in the decision-making process would promote improved personalized care.

## Key points

- Complexity and implications of facial gunshot wounds: Facial gunshot injuries are complex, posing challenges for both medical professionals and victims. The injuries have significant physical, psychological, and economic consequences, necessitating thorough and comprehensive attention due to potential long-term effects on the patient’s life.

- Reconstructive surgery for improved quality of life: Reconstructive surgery yields satisfactory aesthetic and functional outcomes, enhancing patients’ quality of life. By addressing physical damage, it restores appearance and function, positively impacting overall well-being.

- Thorough evaluation and personalized approach: Facial gunshot wound cases can involve unforeseen complexities beyond initial assessments. Comprehensive evaluation is crucial for devising personalized surgical strategies that consider each patient’s unique circumstances.

- Advocating for medico-legal consideration and personalized care: The case report underscores the importance of integrating effective facial reconstruction in medico-legal assessments. A new classification system and personalized evaluation methods can improve care, accounting for treatment costs, expected outcomes, and long-term implications. Tailored care enhances patients’ well-being and decision-making processes.

## Introduction

Every year, more than 32,000 people die and over 67,000 people are injured by firearms in the USA. The mortality rates are highest for self-inflicted firearm injuries, followed by injuries related to aggression [1]. In this context, maxillofacial gunshot wounds (GSWs) result from a wide variety of firearms and projectiles. The actual tissue damage is determined by the mode of energy release during the bullet-tissue interaction and the biological characteristics of the tissues involved, as well as the firing distance [2].

Patients with GSWs require undoubtedly complex reconstruction that should proceed in stages. Firstly, an emergency assessment should be conducted, initiating a multidisciplinary trauma management approach. However, neurosurgical and ophthalmological emergencies must take priority over reparative procedures [2, 3].

It is worth noting that maxillofacial surgery in destructive injuries can yield excellent reconstructive outcomes [4]. Effective reconstruction of devastating wounds can have significant implications both for the survivor’s quality of life and for the level of compensation offered due to firearm-related injuries. Assuming that successful facial reconstruction can influence the extent of compensable damage, it can lead to cost containment regardless of the identified liability profile in the specific case [5].

The present case involves a severe firearm injury resulting in the complete destruction of the face. Following patient stabilization, a meticulous and thorough reconstruction was performed, yielding excellent results. In the light of the forensic relevance of gunshot wounds to the face, as demonstrated by cases of this type, we will consider the role of reconstructive surgery in the assessment of damage and how this should be considered both in terms of success, including the impairment evaluation of the subject afterwards, and cost containment.

### Case report

The case involves a 66-year-old man who was found at home by his wife after a suicide attempt using a Franchi 12-gauge pump-action shotgun. The ammunition used was a Winchester single-round ball (commonly used for boar hunting). The man had a known history of depression and had expressed suicidal intentions prior to the event. The patient was promptly assisted and transported to the emergency department.

**Fig. 1:**
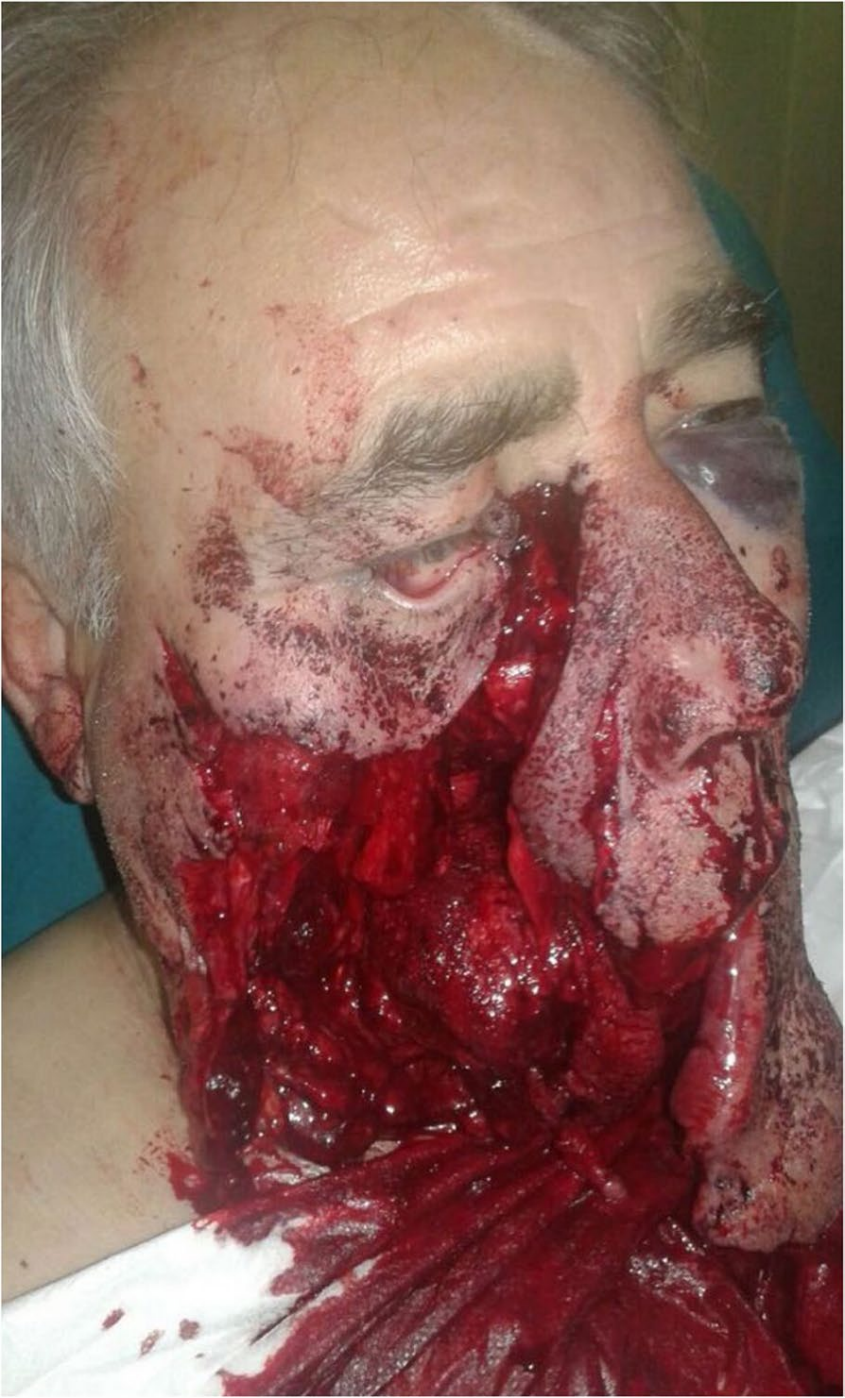
Ballistic trauma with extensive loss of bone and soft tissue and devastation of the oral cavity, mandible, right orbital cavity, and nasal-zygomaticomaxillary complex

As evidenced by the photographic documentation taken on admission to hospital, the gunshot was fired from below, on the right side of the face, resulting in a large contused and lacerated wound with significant tissue loss and multifragmentary fractures of the facial bones (Fig. 1). Vital signs in the emergency department showed a blood pressure of 130/75 mmHg, an oxygen saturation level (SpO_2_) of 94%, and a heart rate of 100 beats per minute.

**Fig. 2:**
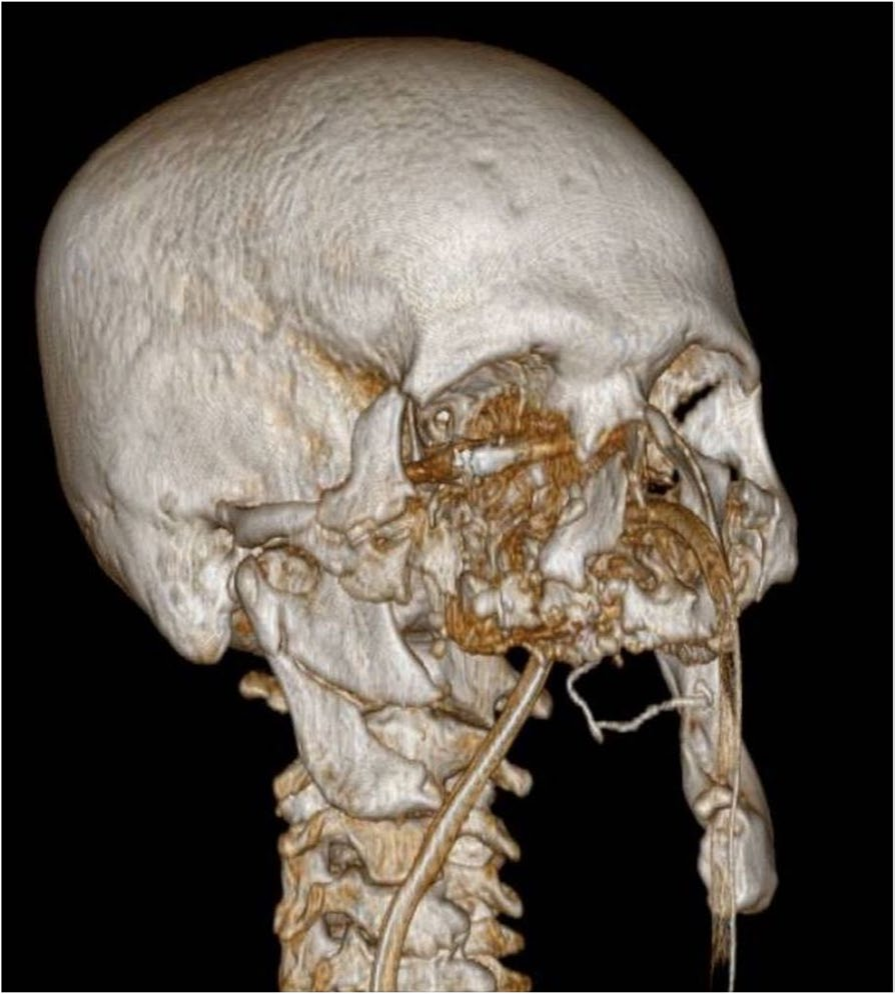
Reconstructions performed on the facial skeleton with a bone window revealed a fracture of the right mandibular condyle with involvement of the mandibular angle and loss of bone substance extending to the left hemimandible from the lower angle to the symphysis

A computed tomography (CT) scan of the brain, neck, facial bones, and cervical spine was performed using a multilayer spiral technique, both before and after contrast agent injection. The CT revealed a hemorrhagic contused and lacerated lesion in the right frontal region of the brain. The neck vessels did not show any significant ongoing bleeding. Facial bone reconstructions with bone window settings revealed a fracture of the right mandibular condyle, involving the mandibular angle with bone loss extending to the left hemimandible from the inferior angle to the symphysis. There were also comminuted and multifragmentary fractures involving the hard palate, ethmoid bone, orbital lamina, and left maxillary sinus. Additionally, metallic foreign bodies were observed within the right eye cavity, along with bone fragments from the orbital floor (Fig. 2).

Given the condition of the facial bones and the evident respiratory difficulty, the patient was sedated and intubated. Fluid infusion and antibiotic prophylaxis were initiated. Considering the clinical presentation, the patient was urgently taken to the operating room to control the ongoing facial bleeding and perform a temporary tracheostomy.

A post-intubation arterial blood gas (ABG) analysis, with a fraction of inspired oxygen (FiO_2_) at 40%, showed the following results: pH 7.213 (normal range: 7.350–7.450), pCO_2_ 58.3 mmHg, pO_2_ 87.4 mmHg (normal range: 75.0–100.0 mmHg), ctHb 8.6 g/dL (normal range: 12.0–18.0 g/dL), FO_2_Hb 92.5% (normal range: 94.0–97.0%), and cLac 8.3 mmol/L (normal range: 0.3–2.5 mmol/L).

Five days after the initial admission, the patient’s general condition stabilized and surgical reconstruction of the facial bones was carried out with three-dimensional modeled titanium reconstruction plates and meshes. Soft tissue reconstruction was achieved using local flaps. The extensive lacerated-contused wounds provided access to the damaged right mandible and orbito-malar region, which had experienced significant bone loss. Mandibular reconstruction was performed using a plate, and the orbito-maxillo-malar and nasal regions were reconstructed using appropriately modeled and fixed titanium meshes and screws. Necrotic skin flaps were excised, and skin flaps were created. Sliding flaps were used to fill the cutaneous deficits.

**Fig. 3:**
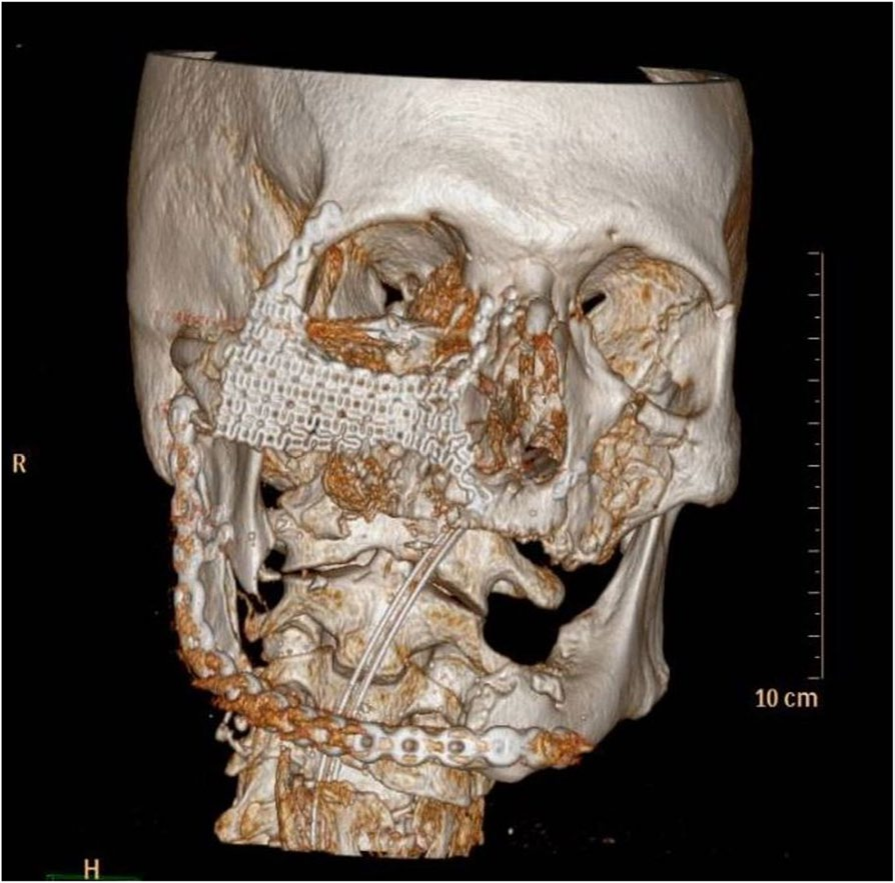
Reconstructions performed on the facial skeleton showed the presence of fixation devices placed during the reconstructive surgical procedure

A postoperative CT scan was performed to confirm the presence of synthesized materials (Fig. 3). The postoperative course was uneventful. The tracheostomy was removed 3 days after the surgery, and oral feeding was gradually reintroduced in the days that followed without oral cavity infection or functional swallowing issues. The patient was discharged from the hospital 10 days after the surgical intervention.

**Fig. 4:**
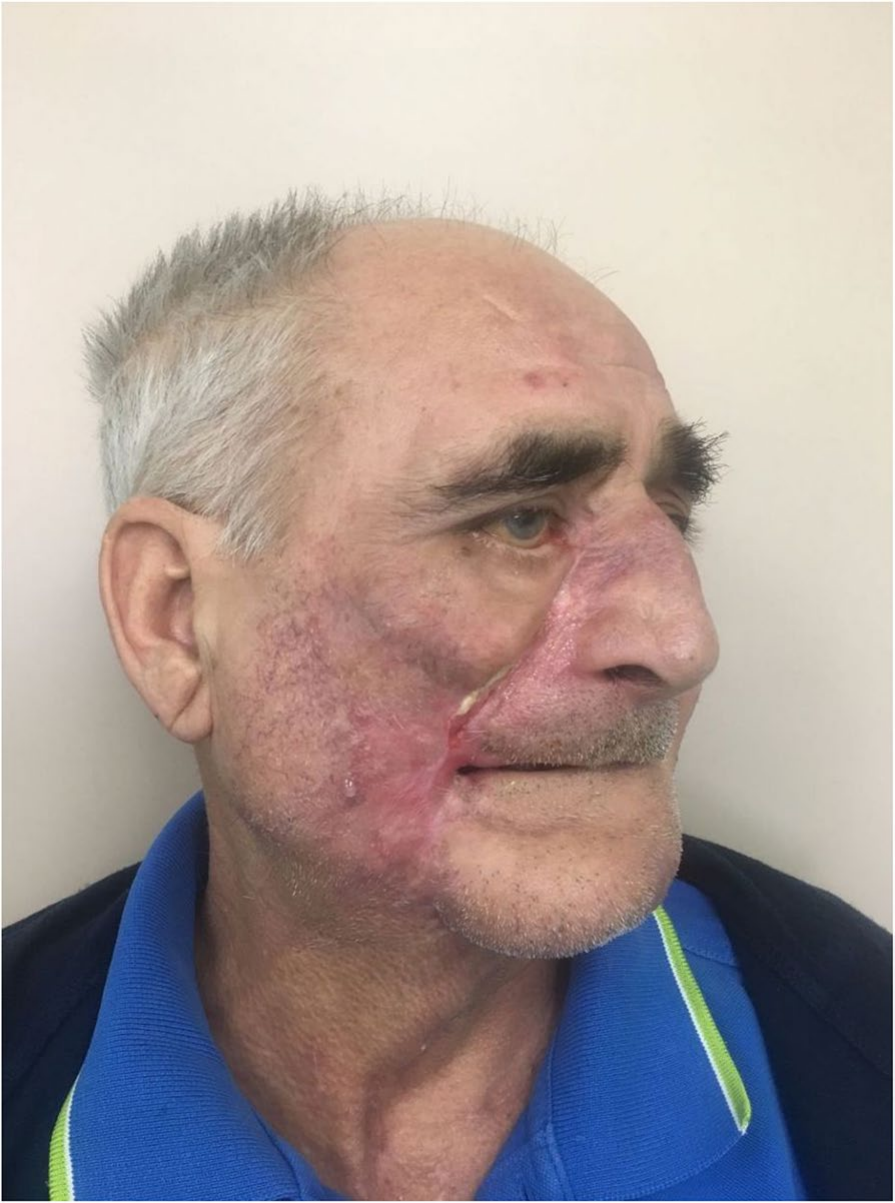
Six-month follow-up visit after reconstruction; the relative provisional nature of the results is due to the fact that no definitive reconstruction was performed, as requested by the patient

Follow-up visits in subsequent weeks showed regular healing and gradual stabilization of scar outcomes and functional outcomes. Six months post-surgery, despite satisfactory wound healing, the patient was offered a facial reconstruction procedure using microvascular free flaps to achieve better aesthetic and functional restoration of the face and facial skeleton (Fig. 4). However, the patient declined the proposed intervention, expressing satisfaction with the results that had already been achieved [6].

### Therapeutic strategies

The initial management of maxillofacial ballistic traumas in this case followed the principles of the advanced trauma life support (ATLS) (protocol, with the immediate implementation of lifesaving procedures and organ preservation (oro-tracheal intubation, hemostasis control, and hemodynamic stabilization). Following ATLS guidelines, once hemodynamic stability was achieved, a multidisciplinary assessment involving emergency specialists and maxillofacial surgeons was conducted. In such cases, a multidisciplinary approach necessitates appropriate imaging, preferably through reconstruction as performed in this instance (Fig. 2) [7].

The initial surgical approach in the acute phase should adhere to the principles of damage control surgery, which involves minimizing surgical procedures in seriously compromised patients to promote physiological recovery in the short term rather than anatomical reconstruction [8, 9].

In similar cases, early and aggressive wound and soft tissue debridement are considered essential to prevent infections and tissue necrosis, along with temporary stabilization of facial bone fragments. This approach is immediately beneficial in reducing pain and bleeding, as well as preventing excessive loss of soft tissue and bone tissue to facilitate subsequent reconstructive surgery [6].

In this regard, there are controversies in the literature regarding the timing of reconstructive surgery, with some studies advocating for the greater effectiveness, in terms of aesthetic and functional restoration, of early primary reconstructive surgery performed as soon as possible compared to delayed secondary reconstruction over time [6, 10].

In the presented case, given the significant loss of soft and bony tissue and the extensive cutaneous and mucosal exposure of the bony structures, the decision was made to proceed with early stabilization and reconstruction using titanium facial skeleton prostheses and soft tissue approximation flaps.

It is believed that this approach limits the risk of infection and tissue necrosis, supporting the patient’s rapid local healing and preserving tissues (quantity and quality of soft and bony tissues) conducive to a subsequent reconstructive phase (via free flaps).

Indeed, the aesthetic results obtained, as evidenced by the comparison between Figs. 1 and 4, support this approach as more functional in avoiding complications and worse functional and ethical outcomes.

Appropriate surgical management in the initial stages of treatment for ballistic facial traumas is of paramount importance, not only for the patient’s life but also for optimal aesthetic and functional outcomes over time. Even any secondary surgery can be severely compromised by delayed or incomplete primary surgery [11, 12].

## Discussion

Gunshot injuries are a relatively infrequent issue, except in certain geographical areas. Following a gunshot wound, patients may experience injuries to the underlying craniofacial skeletal structures, compromised airways, intracranial injuries, and injuries to major blood vessels, all of which may necessitate urgent surgical intervention.

In the case under study, an initial phase of patient stabilization, particularly focusing on respiratory support, was followed by reconstructive surgery of the face. In the initial operative phase, exploration, debridement, and repair of soft tissues and/or facial fractures were performed. Bone stabilization was achieved using plates and maxillo-mandibular fixation. Local or regional flaps were also utilized for less severe injuries. Larger tissue grafts were transferred once the reconstructive phase had been completed with fixation devices, allowing for a single-stage procedure.

Following the surgery, during a stabilization phase with regular monitoring, a second facial reconstruction procedure was proposed. However, the patient declined the second intervention as he was satisfied with the aesthetic results of the initial surgery and preferred to avoid the potential issues associated with an additional invasive procedure.

In this case, the importance of providing the most accurate first reconstructive phase is highlighted, in an attempt to achieve adequate functional and aesthetic restoration, thereby reducing the need for further rehabilitative interventions.

It is evident that firearm injuries to the face, especially when they are highly destructive, result in a significantly elevated risk of morbidity for survivors. In addition to the severe aesthetic damage, these injuries can cause profound psychological harm. In certain situations, the aesthetic damage alone can constitute a substantial economic burden [12, 13].

Patients with severe deformities requiring multiple interventions endure heavy medical, psychosocial, and financial costs. Many patients are eventually lost to follow-up. However, even when bone fixation and soft tissue reconstruction are delayed, patients can regain substantial function after reconstruction and achieve satisfactory aesthetic and psychosocial outcomes [14].

It should be emphasized that the costs of treating firearm injuries impose a significant financial burden, with approximately half of the costs borne by taxpayers, according to estimations [10]. Indeed, each and every gunshot wound incurs extremely high costs [15, 16].

It is crucial to highlight that costs include not only the immediate expenses incurred by the injured individual but also the potential long-term consequences and permanent impairments resulting from the gunshot wound. This aspect becomes particularly critical in civil law cases involving compensation, such as those related to defective firearms or firearm-related injuries resulting from an assault.

In this context, reconstructive surgery can play a significant role as it has the potential to significantly alter the extent of damage, including the quantification of permanent impairment and the costs associated with the injured individual, such as necessary assistance and ongoing medical treatment.

Furthermore, maxillofacial trauma, such as the injuries described in the present case, can cause objective and subjective changes in facial appearance. Facial disfigurement can act as both a trigger and a maintenance factor for mental disorders, such as anxiety, major depression, and post-traumatic stress disorder (PTSD) [17]. It has also been found that there is a strong correlation between subjective ratings of facial injury severity (how much individuals believe their appearance differs from “normal”) and poor psychosocial adjustment. Obviously, an optimal reconstructive outcome would contain the costs of a neuropsychological rehabilitation program that the traumatized subject would commit to [18].

The focus should be on achieving a stabilized final outcome, regardless of the specific path of facial reconstruction, which may vary depending on factors such as the presence of fractures, tissue loss, and involvement of the central nervous system, which may require multiple reconstructive sessions [19, 20].

It is important to specify that extensive and destructive facial trauma, considering the excellent results described and reported in the literature, should be considered partially remediable. This means that the treatment of such injuries can lead to concrete aesthetic and functional outcomes, which do not completely negate the damage incurred (both in criminal and civil contexts), but rather modify the expected outcomes of a destructive trauma [21].

For instance, consider a reduction in masticatory function compared with complete loss of mastication, or severe aesthetic damage as opposed to extremely severe aesthetic damage. Additionally, repeated infections may be significantly reduced or eliminated altogether with effective facial reconstruction, even though multiple sessions may be required.

In this regard, it is believed that the possibility of effective reconstructions should be considered in the decision-making algorithm for the medico-legal assessment of facial gunshot injuries in civil cases, both in terms of care expenses and final outcomes.

An acceptable aesthetic and functional restoration in complex cases of facial ballistic trauma is possible, in the majority of cases, only through multiple surgical procedures and a multidisciplinary approach involving maxillofacial, ophthalmologic, plastic, and neurosurgical specialists.

Therefore, it may be necessary to introduce a new classification system for destructive facial injuries that takes into account the specific circumstances of the case and the realistically achievable results, in a context of personalized assessment.

### Influence of acute treatment on the determination of outcomes in terms of impairment

Devastating facial trauma, as exemplified in the case under examination, results in a multifaceted and complex commitment to individual impairment compensation, giving rise to simultaneous types of damage that an effective emergency treatment is believed to partially influence in terms of outcomes.

From an evaluative standpoint, the face and its components serve multiple functions, including safeguarding underlying organs and aiding in swallowing, respiration, and communication. The skin acts as a protective barrier and regulates temperature. Functional disturbances such as sialorrhea and respiratory compromise may result from neurological injuries or disorders. The face is pivotal to personal identity and emotional expression, and facial disfigurement can have significant social, professional, and psychological consequences. The evaluation of permanent facial impairment necessitates considerations of both anatomical and functional alterations, with a specific focus on the loss of structural and functional integrity. Severe facial disfigurement can lead to significant impairment, with specific assessments ranging from 16 to 50% for serious damages. This category, classified under class 4, includes “massive or total distortion of normal facial anatomy with disfigurement so severe that it precludes social acceptance” combined with any mental and behavioral impairmentst [22].

However, it should be noted that facial disfigurement can result in significantly lesser damages, which, for Class 1, can even amount to percentages well below 10% [22].

In this regard, facial disorders resulting from a destructive trauma, similar to the one presented in this case, encompass general disturbances, compromised hearing, vestibular disorders, structural issues, severe physiognomic damage (especially due to resulting scars), respiratory impairments, mastication impairments, impact on olfaction, and even on phonatory function [23–26]. Therefore, destructive facial traumas exhibit numerous individual damage components, each of which can be effectively addressed through reconstruction, leading to a significant reduction that impacts the final damage percentage in each case [26–28].

Considering the presented case and analogous instances described in the literature, where there is a remarkable recovery compared to the initial damage, it would thus be conceivable to postulate a distinct damage category concerning facial damage, encompassing an independent percentage range of limited magnitude, and including all (reduced) dysfunctional outcomes ensured by emergency surgical treatment. This category could thus be named “face impairment due to facial disorders and/or disfigurement with elective emergency treatment for facial reconstruction” as a unified and comprehensive classification. Furthermore, it should be considered that, at present, there are no impairment categories that are inherently comprehensive of the various functional outcomes of complex facial trauma.

## Conclusion

Facial gunshot wounds pose a complex challenge with significant physical, psychological, and economic implications for the patients involved. However, thanks to reconstructive surgery, satisfactory aesthetic, and functional outcomes can be achieved, improving the patient’s quality of life. Despite the high costs associated with the treatment of firearm injuries, including the burden on taxpayers, it is important to consider the treatment options and expected outcomes when assessing the damage suffered by the individual. Reconstructive surgery provides an important means of modifying the extent of the damage and can impact the quantification of long-term costs and permanent impacts. Therefore, the inclusion of reconstructive surgery in the medico-legal decision-making process, both for necessary care and final outcomes, could be considered as an option, promoting a personalized assessment based on the specific case.

The advent of modern emergency techniques aimed at high-performance reconstruction on both aesthetic and functional levels allows us to hypothesize the definition of a new autonomous damage category within the context of various evaluative scales. This category would encompass intermediate severity in percentage terms and comprehensively include the potential, content-related, and dysfunctional outcomes that follow elective reconstructions, such as the one presented in the case study. Further large-scale studies could ultimately determine the specific percentage range of damage attributable to such injuries, as well as the precise definition criteria for this damage category.

## Data Availability

N/A
